# Fermenting the future – on the benefits of a bioart collaboration

**DOI:** 10.1093/femsyr/foae004

**Published:** 2024-02-05

**Authors:** Diethard Mattanovich, Martin Altvater, Özge Ata, Simone Bachleitner

**Affiliations:** Institute of Microbiology and Microbial Biotechnology, Department of Biotechnology, University of Natural Resources and Life Sciences, Vienna (BOKU), 1190 Vienna, Austria; Austrian Centre of Industrial Biotechnology (acib GmbH), 1190 Vienna, Austria; Institute of Microbiology and Microbial Biotechnology, Department of Biotechnology, University of Natural Resources and Life Sciences, Vienna (BOKU), 1190 Vienna, Austria; Austrian Centre of Industrial Biotechnology (acib GmbH), 1190 Vienna, Austria; Institute of Microbiology and Microbial Biotechnology, Department of Biotechnology, University of Natural Resources and Life Sciences, Vienna (BOKU), 1190 Vienna, Austria; Austrian Centre of Industrial Biotechnology (acib GmbH), 1190 Vienna, Austria; Institute of Microbiology and Microbial Biotechnology, Department of Biotechnology, University of Natural Resources and Life Sciences, Vienna (BOKU), 1190 Vienna, Austria

**Keywords:** art, bioart, yeast, biotechnology, science

## Abstract

In this article we explore the intersection of science and art through a collaboration between us scientists and the bioartists Anna Dimitriu and Alex May, focusing on the interface of yeast biotechnology and art. The collaboration, originally initiated in 2018, resulted in three major artworks: CULTURE, depicting the evolution of yeast and human societies; FERMENTING FUTURES, illustrating a synthetic autotrophic yeast and its link to lactic acid production; and WOOD SPIRIT—AMBER ACID, inspired by the VIVALDI project targeting CO_2_ reduction to methanol. We emphasize the reciprocal nature of the collaboration, detailing the scientific insights gained and the impact of artistic perspectives on us as researchers. We also highlight the historical connection between art and science, particularly in the Renaissance periods, and underscore the educational value of integrating art into science not only to support public engagement and science dissemination, but also to widen our own perceptions in our research.

## Teaser

Fermenting the Future is a summary of our experiences and perspectives of our bioart collaboration and the resulting artworks, addressing the cultural significance of yeast biotechnology while also highlighting the inspiring relationship between art and science throughout history.

## Introduction

The connection between science and art is multifaceted and has been a subject of exploration and discussion for centuries. It is characterized by shared interests such as observation, creativity, and the quest for exploring and understanding the unknown. Art and science have often inspired each other, but more than that, the direct transfer of scientific knowledge or art practice has enabled breakthroughs at certain moments in history, changing future practice.

Peter Weibel, the former director of the Center for Art and Media (ZKM) in Karlsruhe, Germany, claimed that the renaissance periods have created specially vibrant scenes of art-science collaborations (Weibel 2023). The exhibition Renaissance 3.0 in the ZKM defines three renaissance periods: the Arab Renaissance, the Italian Renaissance with Florence as its nucleus, and the modern, potentially currently happening Renaissance, largely driven by media art and bioart. Most prominently, the Florentine Renaissance has been discussed intensely at the interface of art and science for its incentives to spark collaborations of artists and scientists (Puett and Puett 2016), with some stellar figures producing great achievements in both areas, often referred to as polymaths, skilled individuals educated in a number of different subjects. By refusing barriers and limitations in scientific methods, fields of studies, and the integration of art in knowledge gain, Alexander von Humboldt is often regarded as the last European polymath and “Renaissance man” (Gunderman 2014). As a bridge to the present, today, polymathy is considered a prevalent trait of Nobel laureates, many of them even describing polymathy as an intentional choice to optimize creative potential (Root-Bernstein and Root-Bernstein 2023). Intriguingly, using polymathy as educational tool by teaching STEMM (science, technology, engineering, mathematics, and medicine) students arts, crafts and design practices can significantly improve learning outcomes proving the strong correlation between art and science (Root-Bernstein et al. 2019).

These records build on analyses of the past and their interpretations towards a future that is emerging now. Over the past few years our lab has developed a deep interest in the interface of biosciences and art, often referred to as bioart. This term was introduced to define the art movement where artists are working with living (micro)organisms or use processes of life to create their artwork. In the following we summarize experiences and learnings from that collaboration, with an outlook to future areas to be explored together.

## Our part in bioart

In 2018, we have initiated a collaboration with bioartist Anna Dumitriu and digital artist Alex May, to transform our research on yeast biotechnology to art, with the initial intention to exhibit the artwork and the process at the International Congress on Yeasts, planned for 2020 in Vienna (Dumitriu et al. 2021). Initially, we planned to invite the artists for a residency in our lab to create some yet undefined artworks to show them at the congress location in an area open to the public. The entire endeavor has grown much larger since then, including collaborations with curators, gallerists and contemporary art historians, bringing the artwork, the scientific background and our collaborative approach to major art galleries in Europe. Premiering at the Vienna Künstlerhaus, AT, parts of the artwork were then shown in Kunsthaus Wiesbaden, DE, The North Wall in Oxford, UK, Spazju Kreattiv in Valletta, MT, Bastion Maria Theresia in Timișoara, RO, ZKM | Center for Art and Media in Karlsruhe, DE (as part of Renaissance 3.0), and at the Nobel Prize Museum Stockholm, SE. Especially the last two locations refer intriguingly to the connections of bioart with Renaissance periods and polymathy, as briefly discussed in the introduction.

While initiating the collaboration without a distinct intention besides enabling the creative process and presenting the outcome, we have realized remarkable feedback on our science and technology, clearly demonstrating that this collaboration is not a one-way from scientific knowhow and stories, technologies and materials towards the realization of the artwork.

In the following, our collaboration, our perceptions and learnings from it, and feedback to us as scientists will be explained with three examples, by means of three major artworks that emerged from the collaboration.

### Culture

CULTURE tells a story of evolution of yeast and human societies. We humans see ourselves as a species that is able to change nature around us by impacting the environment of other life forms for our purpose. At the same time we tend to neglect the ability of other species to do so as well, however yeast also might have played a similar role during the neolithic era. Its ability to ferment carbohydrates to ethanol and carbon dioxide has inspired humans to brew beer and bake leavened bread, inducing a demand for cereal crops which, over time, motivated them to settle and cultivate crops (Dietrich et al. 2019). The earliest indications of very first human settlements, connected with brewing and baking, are found in Göbeklitepe in Turkey (Dietrich et al. 2012) and Raqefet Cave in Israel (Liu et al. 2018).

**Figure 1.**
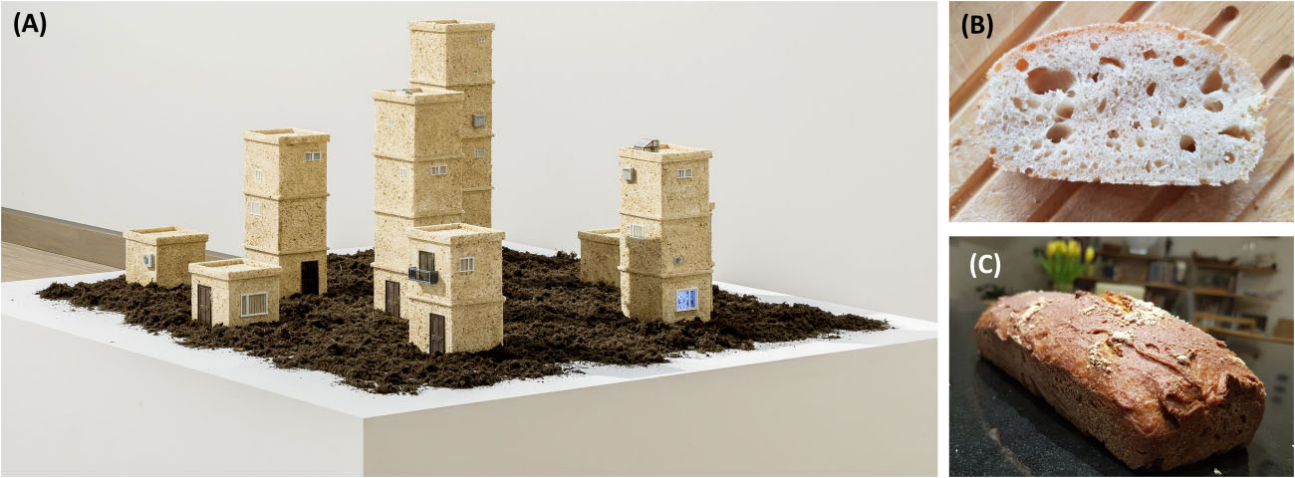
(A) CULTURE installation symbolizing the connection of yeast's fermentation evolution and cultural evolution of human throughout the history. © Left Studio, Vienna. (B) A “*Pichia*” bread fermented by the *CRA1* overexpressing strain. (C) Baked bread using whole-grain Einkorn wheat, one of the oldest domesticated wheat species, and leavened with the fermenting *K. phaffii* strain. This bread loaf represents bread baked by the first human settlers in the Fertile Crescent in the Middle East, and was crushed to cover the sculpture.

Discussions on the historical connections of humans and yeasts were initiated by research in our lab on the evolution of fermentation in yeasts. Some years ago we found a genetic master regulator of glycolysis and fermentation in *Komagataella phaffii* (formerly known as *Pichia pastoris*) (Ata et al. 2018), and we investigated its potential role in the evolution of fermentative traits in yeasts. The artists planned to span an arc from the prehistoric connections of yeasts and humans, mirroring the cultural evolution of human settlement with the metabolic evolution of yeast fermentation. These detailed discussions have definitely changed our scientific perspective on our object of study, yeasts. With the first attempts to bake bread with this engineered potential “missing link” of yeast metabolic evolution we realized that it can ferment glucose, but not maltose, the key structural subunit of starch, and it could not leaven bread. In *Saccharomyces cerevisiae* maltose is transported into the cell where an intracellular maltase hydrolyzes maltose into glucose. Here, we used another approach and expressed an extracellular maltase using marker-free CRISPR-Cas9 technology. We could demonstrate the success of strain engineering as we could bake bread with the new *K. phaffii* strain, which was then used as a material for the creation of the sculpture (Fig. 1).

### Fermenting Futures

**Figure 2.**
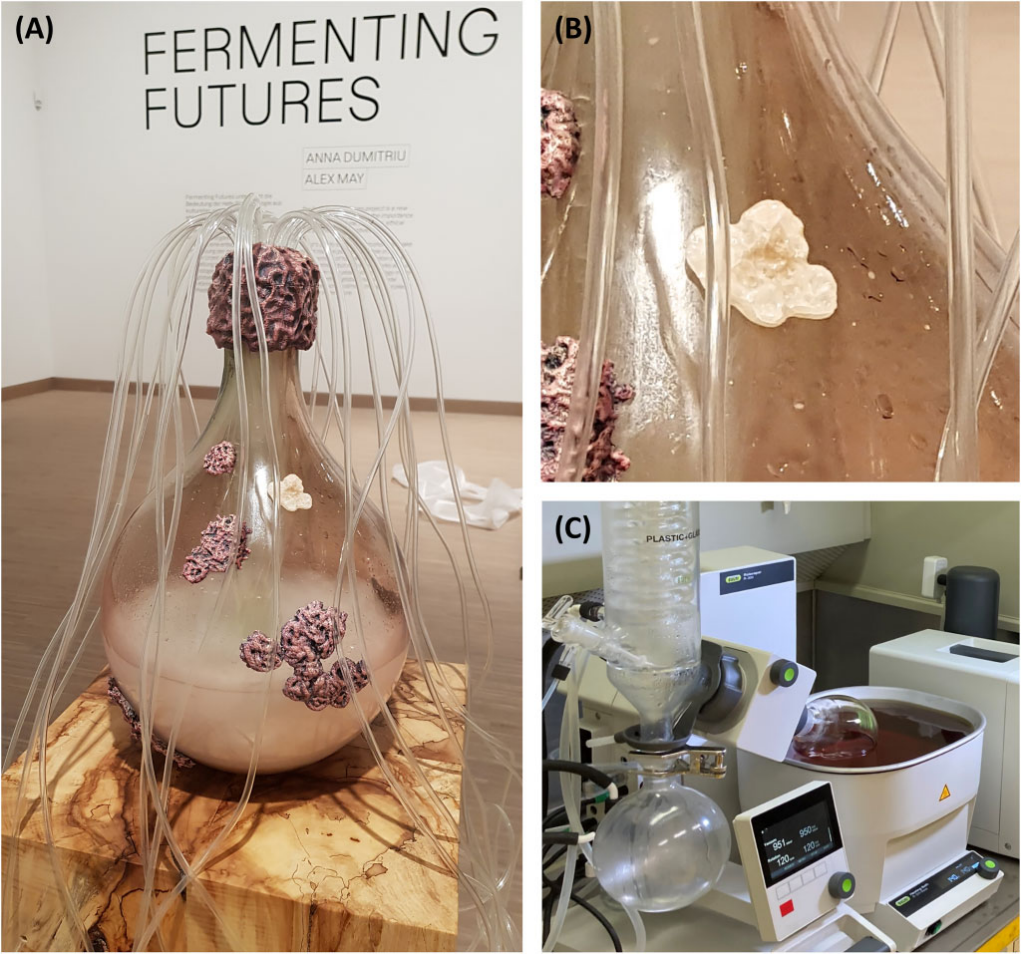
(A) FERMENTING FUTURES sculpture resembling a laboratory bioreactor containing lactic acid-producing yeast. (B) Closeup of a PLA-form containing yeast-produced lactic acid. (C) Technical setup including a rotation evaporator for polymerizing lactic acid to PLA.

Some years ago, we created a synthetic autotrophic yeast by re-engineering *K. phaffii* to assimilate CO_2_ instead of methanol (Gassler et al. 2020). After further metabolic engineering this strain can also produce organic acids (as chemical building blocks) by the conversion of CO_2_. A special attention went to lactic acid as the monomer of polylactic acid (Baumschabl et al. 2022). For the narrative of the sculpture FERMENTING FUTURES we connected this work with another project where we produce lactic acid from glucose with engineered *S. cerevisiae*. The sculpture is composed of a glass vessel connected with a bundle of silicon tubes, resembling a bioreactor where the yeasts are cultivated in the laboratory. 3D printed models of yeast colonies cover the flask, one of them made of the biodegradable polymer polylactic acid (PLA) that was polymerized in our laboratory containing the yeast-produced lactic acid (Fig. 2). The vessel rests on a plinth of chestnut wood—the tree species where the first isolate of *Pichia pastoris* (now named *Komagataella spp*.) was found. A major intention of the artists was to produce PLA in the lab from a mix of lactic acid produced from glucose but notably also lactic acid made from CO_2_ with the autotrophic strain. We had an interest in this polymerization process but as we are no polymer chemists we had refrained from practically implementing it. In retrospect, we have probably avoided the hassle of engaging in a technology we felt uncomfortable with, being non-experts. As there was a need now but no protocol in place, we consulted our colleagues and assembled several methods (Moon et al. 2000, Auras et al. 2010) to adapt them to our needs and technical setup for the successful polymerization of lactic acid to PLA. Thus, we learned a lot about the polymerization technology adjacent to our own in the process chain.

The plinth supporting the sculpture was to be made of horse chestnut wood—the tree species where the first sample of *K. phaffii* was isolated from. The literature describes it as the edible chestnut (*Castanea sativa*), however a look at the original paper by A. Guilliermond (1920) appeared appropriate. It revealed a translation error that was transcribed further until recently: Guillermond describes the tree as a *marronnier* which is tempting to be translated as the edible chestnut (which is, however, called *châtaignier* in French). Having revealed this little mystery of the origin of the yeast we had to solve the puzzle where to get horse chestnut wood from, which is not traded, different to the edible chestnut.

### Wood spirit—Amber acid

The third artwork discussed here was inspired by the European Union Horizon 2020 project VIVALDI (grant number 101000441) that aims to reduce CO_2_ of industrial off-gas electrochemically to methanol, and to upcycle methanol to organic acids with the metabolic activity of *K. phaffii*. Succinic acid is one of the target products of VIVALDI. It is known as a base chemical for organic synthesis that can be converted into a variety of products. Anna Dumitriu was inspired by the traditional names of substrate and product to name the artwork WOOD SPIRIT—AMBER ACID: methanol was first produced by dry distillation of wood, and succinic acid was first prepared by distillation of amber. Wood is also a likely source of methanol for the yeasts living on it.

Besides the definition of sources of the substrate, we had to answer the application of succinic acid in chemical technology. Succinic acid is often described as a base chemical, and applications for polymers, resins and solvents are indicated. The artist, however, asked for defined products that are made of succinic acid, to select material for the artwork from that. This made it evident to us, although having a rough picture of the potential application of our target product, that we have not taken the time to research its actual use in detail. One quick answer is nylon: many types of nylon (or polyamides) are produced, defined by the types of precursors (dicarboxylic acids + diamines, or aminocarboxylic acids), and the chain lengths of these monomers. Succinic acid is a precursor of nylon PA 5.4, where the 4 denotes the chain length of the acid precursor.

Questions continued for other products out of succinic acid, and after some further research we found two rather new types of synthetic pigments which were developed in the 1970 s and introduced to artists’ palettes recently: quinacridone pigments, featuring golden to magenta hues, and the diketopyrrolopyrrole family with a colour range from orange to violet (Christie and Abel 2021, 2022). Succinic acid, as many other potential products of industrial biotechnology, is a business-to-business product not directly known to end consumers. We have to acknowledge that we as researchers working on the production of the precursors were not familiar with the details of the application, and the research for the artwork taught us a lot beyond our area of expertise.

**Figure 3.**
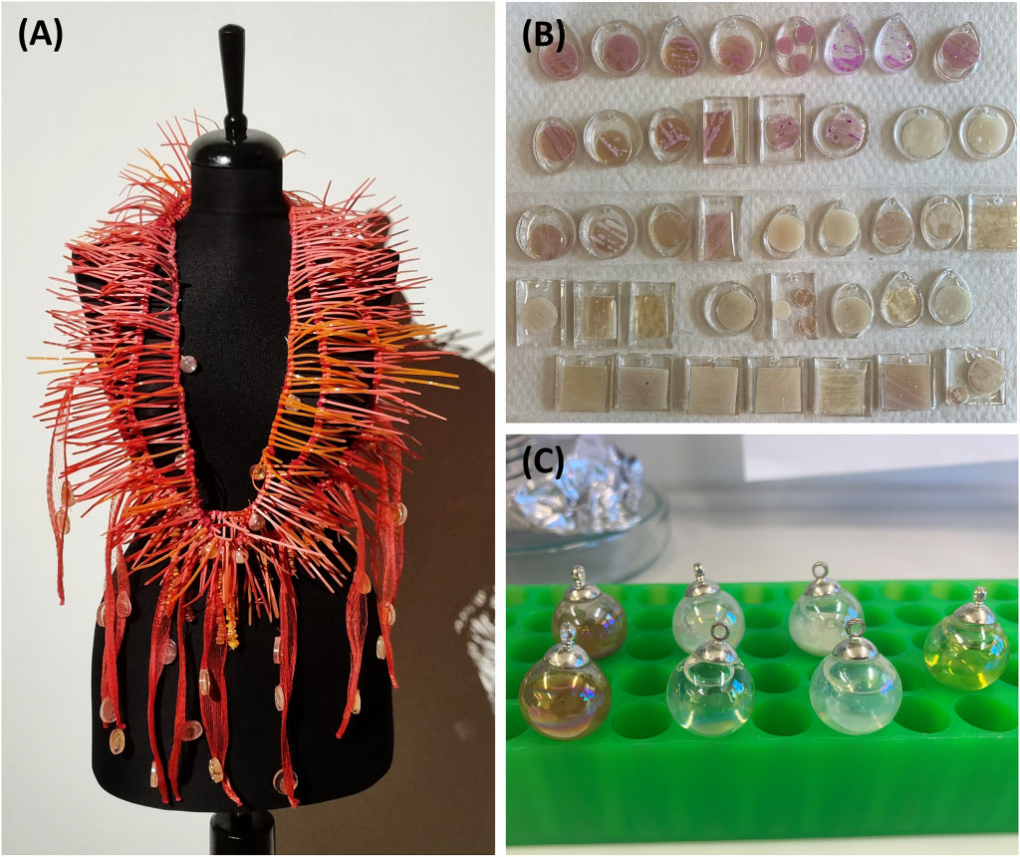
(A) WOOD SPIRIT—AMBER ACID is a sculptural necklace narrrating the story of the sustainable production of succinic acid from methanol using *Komagataella phaffii*. (B) Pendants containing engineered yeast cells that were embedded in epoxy resin. (C) Glass beads were filled with fermentation supernatants containing yeast cells and succinic acid.

The artwork is a necklace made of nylon that was dyed with quinacridone pigments, including some amber beads and pendants containing culture liquids and yeast colonies (Fig. 3). A positive side effect of preparing these pendants was learning how to conserve agar cultures for visual inspection by embedding them in epoxy resin, a technique we are using now also for science fairs.

## Conclusions

Crossing these boundaries between art and science and following the creation of an artwork, was not only fun and exciting for us as researchers, but encountered us also with new experimental techniques, approaches and mindsets. Despite having obvious differences, through this collaboration we experienced the numerous similarities of approaching art and science. During the actual research, we scientists are guided by our experimental outcomes, and data must be evaluated as objectively as possible, while art making and perception can be much more emotional, subjective and influenced by opinions. But at the same time, passion and curiosity of exploring the world, creating new knowledge, concepts, and ways of thinking are the foundations and driving forces of both art and science. Revealing the scientific truth about yeasts makes us scientists as excited or mesmerized as seeing an art piece. Furthermore, being involved in a bioart project, where art and science truly meet, created a deeper understanding of our research and expanded our perspective and knowledge about the yeast world and especially the connection between yeasts and humans.

Supporting the artistic process was not the end of our interest but we were keen to see what kind of emotions and perspectives the resulting artworks would spread. As the outcome of our collaboration grew much larger than anticipated and resulted in a broad wave of positive public response, we came in touch with the art museum scene and the organization of exhibitions, and we were involved in science talks, visitor discussions and guiding tours through the art show. Engaging with visitors was more important as we did not aim to advertise our research or bias the artists with our way of thinking. Just as hoped for, we could observe the impact of the collaboration both on our research, and on the artwork unfold by different modes of exhibition, approaching visitors in a variety of different ways.

In addition, this transdisciplinary collaboration pushed our work in new directions including the search for new materials and techniques, or by just gaining a new perspective on our own research. However, it also opened new and important questions concerning the display of bioart and the possible contributions of the biosciences to that: can bioart be living and/or deteriorable, or should it be stable and “dead”, to be suited for museums? How can bioart be conserved and regenerated, and is this desired at all? Will there be a development of a bioart material canon, based on either living, inactivated, or dead material? These are just some topics for new collaborations where life sciences will have a key role.
